# Urban Pigeons (*Columba livia*) as a Source of Broad-Spectrum β-Lactamase-Producing *Escherichia coli* in Lisbon, Portugal

**DOI:** 10.3390/antibiotics11101368

**Published:** 2022-10-06

**Authors:** Samanta Freire, Teresa Grilo, Laurent Poirel, Marta Aires-de-Sousa

**Affiliations:** 1 Laboratory of Molecular Biology, Portuguese Red Cross, 1600-680 Lisboa, Portugal; 2 Medical and Molecular Microbiology Unit, Faculty of Science and Medicine, University of Fribourg, 1700 Fribourg, Switzerland; 3 INSERM European Unit (IAME, France), University of Fribourg, 1700 Fribourg, Switzerland; 4 Swiss National Reference Center for Emerging Antibiotic Resistance (NARA), 1700 Fribourg, Switzerland; 5 Escola Superior de Saúde da Cruz Vermelha Portuguesa-Lisboa (ESSCVP-Lisboa), 1300-125 Lisboa, Portugal; 6 Laboratory of Molecular Genetics, Instituto de Tecnologia Química e Biológica António Xavier (ITQB), Universidade Nova de Lisboa (UNL), 2780-157 Oeiras, Portugal

**Keywords:** pigeons, ESBL, *Enterobacteriaceae*, *Escherichia coli*, Portugal

## Abstract

Wild birds may be healthy carriers, and therefore, may be involved in the dissemination of clinically relevant antimicrobial-resistant bacteria, such as extended-spectrum β-lactamases (ESBL) and carbapenemase-producing *Enterobacteriaceae*. This study evaluated whether urban pigeons living in five spots in Lisbon, Portugal, may be colonized and, therefore, constitute potential spreaders of multidrug-resistant bacteria. A total of 100 pigeon fecal samples were collected in different urban areas for the detection of ESBL- or carbapenemase-producing *Enterobacteriaceae*. All β-lactamase-producing isolates were tested for antimicrobial susceptibility and their genetic backgrounds were characterized by multilocus sequence typing. Of the 100 fecal samples collected, nine ESBL-producing *Escherichia coli* (9%) were identified. Three isolates carried the *bla*_CTX-M-15_ gene, three isolates harbored the *bla*_CTX-M-27_ and three isolates carried the *bla*_SHV-12_ gene. Genotyping of the nine ESBL-producing *E. coli* strains revealed seven different sequence types (STs) including ST10, ST131, ST154, ST206, ST1488 (SLV ST10), ST2858 and ST3576, most of which have been already described in humans, animals or in the environment. Urban pigeons constitute a potential source of ESBL genes and may be a transmission vehicle of multidrug-resistant bacteria in the environment.

## 1. Introduction

*Enterobacteriaceae*-producing extended-spectrum β-lactamases (ESBL) and/or carbapenemases, which were initially exclusively detected in the hospital setting, are now frequently found in the community among domestic and wild animals, and also in the environment. The localization of ESBL- and carbapenemase-encoding genes on mobile genetic elements that can be transferred between different bacteria and hosts, has turned ESBL- and carbapenemase-producing *Enterobacteriaceae* to a One-Health issue.

Wild birds are well known to be carriers of antibiotic-resistant bacteria and antibiotic-resistant genes [1]. Birds living in proximity to human settings may acquire antimicrobial-multiresistant bacteria from anthropogenic sources, such as urban wastewater and landfills, and therefore, act as sentinels, mirroring human activity [2]. Conversely, colonized animals may be sources of such resistant bacteria, eventually leading to acquisition by humans. The first antibiotic-resistant bacteria reported in wildlife (*Escherichia coli* is resistant to chloramphenicol) were isolated in pigeons around 1975 [3]. Subsequently, many other bird species have been found to carry antibiotic-resistant *E. coli*, including ducks, geese, cormorants, birds of prey, gulls, doves, vultures and passerines [4]. In addition, wild birds play an important role in the spread of antibiotic resistance through the ability to migrate long distances [5].

The population structure of ESBL-producing *E. coli* is dominated globally by the high-risk clone belonging to sequence type (ST)131. Other *E. coli* STs associated with ESBLs are ST10, ST38, ST131, ST315, ST393, ST405 and ST648 [6].

During recent years, several reports indicated the presence of ESBL- and carbapenemase-producing bacteria among different species of urban and wild birds [1,7,8,9,10,11]. In Portugal, we reported alarming levels of ESBL- (55%) and carbapenemase- (16%) producing *Enterobacteriaceae* in gull fecal samples [12]. However, no studies evaluated the role of Portuguese urban pigeons as reservoirs of ESBLs and carbapenemases.

Therefore, the aim of the present study was to evaluate the intestinal carriage of *Enterobacteriaceae*-producing ESBL and/or carbapenemases in urban pigeons in different spots in the Lisbon area, and to characterize the collected isolates.

## 2. Results

**Figure 1:**
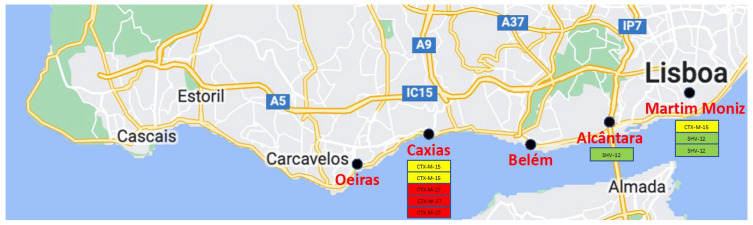
Map depicting the different sampling sites and the detected ESBLs.

From the 100 pigeon fecal samples inoculated on the different selective media, nine enterobacterial isolates (9%) were recovered, showing a phenotypic antibiotic resistance profile compatible with the production of ESBLs. These isolates were subsequently confirmed to be *E. coli* ESBL producers. There were no isolates growing on the selective media for carbapenem or colistin resistance. ESBL-producing isolates were found in samples from three out of the five sampling sites, and predominantly from Caxias (Table 1 and Figure 1).

Most of the ESBL-producing isolates showed decreased susceptibility to temocillin and ceftazidime (100%), aztreonam (*n* = 8; 89%) and cefotaxime (*n* = 6; 67%). In addition, some of those isolates showed resistance to tetracycline (*n* = 4; 44%), SXT (*n* = 3; 33%), ciprofloxacin, gentamicin and tobramycin (*n* = 2 each; 22%). None of those ESBL-producing isolates presented resistance to amoxicillin/clavulanic acid, cefoxitin, ertapenem, imipenem, ceftazidime/avibactam, amikacin and fosfomycin. Therefore, despite being ESBL producers, these isolates are not highly resistant, displaying no resistance to amoxicillin/clavulanic. 

The genotypic characterization identified three different ESBLs (Table 2), with mainly CTX-M enzymes in group 1 (6/9; 66%). Three isolates carried the *bla*_CTX-M-15_ gene, three isolates harbored the *bla*_CTX-M-27_ gene, and the three last isolates carried the *bla*_SHV-12_ gene. The SHV-12- and CTX-M-15 producers were recovered from two different sampling sites, while CTX-M-27 was exclusively found in Caxias (Figure 1), without statistical significance.

The genotyping of ESBL-producing *E. coli* strains revealed seven different STs including ST10, ST131, ST154, ST206, ST1488 (SLV ST10), ST2858 and ST3576 (Table 2). The *E. coli* ST10, ST154 and ST3576 carried the *bla*_SHV-12_ gene, while ST206 and ST2858 harbored *bla*_CTX-M-15_, and *bla*_CTX-M-27_ was associated with ST131 and ST1488. 

## 3. Discussion

Our study revealed the occurrence (9%) of ESBL-producing *E. coli* colonizing urban pigeons in Lisbon, Portugal. Few studies have been conducted on urban birds in Portugal and our study was the first to investigate antibiotic-resistant bacteria in pigeons. As compared to international studies, lower rates of ESBL-producing *E. coli* among pigeons were reported in France (1/71; 1.4%) [13], Brazil (3/107; 2.8%) [14], Bangladesh (7/150; 4.7%) [15] and Algeria (18/276; 6.5%) [16]. This variation may reflect differences in feeding habits or immune status [17].

In a previous study that we recently conducted, a frequent occurrence of carbapenemase- and ESBL-producing *Enterobacteriaceae* (16% and 55%, respectively) was identified among gulls using samples collected from the Lisbon coastline [12], contrasting with much lower rates in pigeons (0% and 9%, respectively) in the present study. Interestingly, the single sampling site common to both Portuguese studies (Caxias), showed the highest rates of ESBL-producing isolates in pigeons and gulls (25% and 83%, respectively). In this particular sampling site, there is an effluent with a previous history of coliform contamination due to discharges of rainwater of an urban origin and clandestine domestic wastewater discharges [18], which may explain the higher gut colonization rates among pigeons and gulls that may drink the contaminated water. Noteworthy, Ngaiganam et al. also observed that ESBL-producing *Enterobacteriaceae* were more frequently isolated from beach gulls than from pigeons [13].

Environmental water contaminated by human gut colonizers may be the major source of intestinal colonization of urban birds. Indeed, most of the ESBL-producing isolates were recovered from birds living close to an urban effluent. As observed with gulls, isolates colonizing pigeons showed significant molecular features with human isolates. Indeed, the three types of ESBLs identified in this study (CTX-M-15, CTX-M-27 and SHV-12) are frequently found among humans (sick and healthy) in Portugal [19,20]. In a study that evaluated the intestinal carriage of ESBL-producing *Enterobacteriaceae* in admissions at a hospital in Lisbon, CTX-M-15 was the predominant ESBL (50%), followed by CTX-M-27 (27%) and SHV-12 (6%) [20], correlating with the ESBL types observed among pigeons. Of note, CTX-M-15 is also the main ESBL reported worldwide among birds, together with CTX-M-1 [21].

Except for ST154 and ST2858, the remaining STs described here (ST10, ST131, ST206, ST1488 and ST3576) have been previously reported. Whole-genome sequencing of a collection of 403 *E. coli* isolates from fecal human samples over 30 years (1980 to 2010) revealed that ST10 was one of the five major lineages among healthy humans in France [22]. ST10 was also found as the predominant clone among *E. coli* recovered from students in Lisbon [23] and was found among healthy and sick cats from the same geographical area as well [24]. ST131, which is the most widespread high-risk clone of multidrug-resistant *E. coli* responsible for infection outbreaks worldwide [25] has been detected in wild seabirds in Brazil [4]. ST206 is mainly associated with animals; it is the main clone (*n* = 35; 69%) among chickens in Nigeria [26], and frequently found among farm animals in China [27]. A single ST1488 *E. coli* isolate, harboring CTX-M-3, has been recovered from a human sample in Hong Kong [28]. 

Moreover, some of the STs found in the present study have been previously reported in the environment. Indeed, ST131 was present in ESBL-producing *E. coli* in reservoir water in Singapore [29], whereas ST10 has been found in antibiotic-resistant *Enterobacteriaceae* circulating in sewage and aquatic environments in Ireland [30] and China [31]. On the other hand, ST3576 has been found in a susceptible *E. coli* isolate recovered downstream of a wastewater treatment plan in a river in the United Kingdom [32]. Therefore, our study is consistent with previous studies in which human, animal and environment isolates shared mainly identical STs. 

Interestingly, none of the ESBL-producing *E. coli* carrying the *bla*_CTX-M-15_, *bla*_CTX-M-27_ or the *bla*_SHV-12_ genes showed resistance to amoxicillin/clavulanic. Contrary to most human isolates, these isolates seem to have a genetic background closer to the wildtype.

The presence of ESBL and carbapenemases genes as well as the *mcr*-type colistin-resistance gene were evaluated using a targeted analysis (PCR). Further genome-wide analysis could provide valuable information of additional genes associated with drug resistance in these samples. Moreover, since this study was conducted in the wild, there are limitations of controlling the experiments, e.g., feeding habits, environmental exposure, etc. Future investigations using pigeons in captivity could overcome this limitation.

In conclusion, this study evidenced that pigeons in urban areas, which live in close contact to humans, may acquire antimicrobial-multiresistant bacteria from anthropogenic sources and play a role as potential reservoirs of multidrug-resistant bacteria, namely *E. coli* ESBL producers. The surveillance and monitoring of antibiotic-resistant *Enterobacteriaceae* remains essential in a One Health approach to counteract the spread of such pathogens.

## 4. Materials and Methods

### 4.1. Sample Collection and Bacterial Isolates

Between September 2021 and May 2022, a total of 100 fresh pigeon (*Columba livia*) fecal samples were collected at five urban areas (20 samples each) in the Lisbon area, Portugal. Sampling sites were chosen in areas with an eventual contamination with multidrug-resistant bacteria: next to the waste disposal in a social neighborhood (Alcântara), next to a hospital (Martim Moniz), in the border of Tejo River (Belém), and near urban effluents (Caxias and Oeiras)—Table 1 and Figure 1. To minimize repeated sampling, the recovery of specimens was performed on the same day for each sampling area and was limited to 20 samples. To prevent cross-contamination, only wet, fresh and separate feces were collected into sterile plastic tubes (Frilabo, Maia, Portugal).

Samples were stored at room temperature and transported within 2 hours to the laboratory where they were incubated overnight in 3 mL of Tryptic Soy Broth (TSB) (Frilabo) for enrichment. The next day, a volume of 25 μL of each broth was inoculated onto three selective media: (i) CHROMagar ESBL (ChromAgar, Paris, France) to select for ESBL producers; (ii) CHROMagar mSuperCARBA (ChromAgar) to select for carbapenem-resistant isolates, and CHROMagar COL-APSE (ChromAgar) to select isolates resistant to colistin. The isolates that were selected using the different media were identified at the species level using the API20E system (bioMérieux, La Balme-les-Grottes, France) or by MALDI-TOF in case of doubtful results. 

### 4.2. Antimicrobial Susceptibility Testing 

Antimicrobial susceptibility testing was performed on all isolates by using the disc diffusion method on Mueller-Hinton (MH) agar plates (Neogen, Lansing, Michigan) for ticarcillin, amoxicillin/clavulanic acid, cefotaxime, ceftazidime, temocillin, cefoxitin, ertapenem, imipenem, ceftazidime/avibactam, aztreonam, ciprofloxacin, trimethoprim-sulfamethoxazole (SXT), tetracycline, amikacin, gentamicin and tobramycin (Bio-Rad Laboratories, Algés, Portugal), following EUCAST recommendations and breakpoint tables. Susceptibility to fosfomycin was evaluated by the disk diffusion method on MH agar plates supplemented with 25 μg/mL of glucose-6-phosphate, according to EUCAST guidelines [33].

### 4.3. Identification of Resistance Determinants

The identification of ESBL genes was performed by PCR as previously reported [34] for all isolates showing a phenotypic antibiotic resistance profile compatible with the production of ESBLs. Isolates showing susceptibility to cefotaxime were first tested for the presence of SHV, while isolates resistant to this antibiotic were tested for CTX-M group 1 and, subsequently, for CTX-M group 9. All positive amplicons were purified using the ExoCleanUp FAST PCR purification kit (VWR, Alfragide, Portugal) and sequenced to identify the ESBL variant (Eurofins Genomics, Ebersberg, Germany). Screening of the *mcr* colistin resistance genes was performed by using previously published primers [35]. 

### 4.4. Molecular Typing

The clonal relationship of the ESBL-producing isolates was evaluated by multilocus sequence typing (MLST), and STs were assigned using the MLST database for *E. coli* (https://cge.cbs.dtu.dk/services/MLST-2.0/, accessed on 23 August 2022). 

## Figures and Tables

**Table 1:** Samples recovered and samples colonized with ESBL-producers.

Sampling Site	Sampling Date	Samples Recovered	Samples Colonized with ESBL-Producers No. (%)
Alcântara (near a waste disposal)	21 September 2021	20	1 (5%)
Martim Moniz (near a hospital)	30 October 2021	20	3 (15%)
Caxias (near an effluent)	17 November 2021	20	5 (25%)
Oeiras (near an effluent)	20 February 2022	20	0
Belém (near Tejo River)	15 May 2022	20	0
Total		100	9 (9%)

**Table 2:** Characteristics of the nine ESBL-producing *E. coli* isolates.

Fecal Sample	Sampling Site	Isolate	ESBL	MLST	TIC	AMC	CTX	CZD	TEM	FOX	ETP	IMP	CZA	ATM	CIP	SXT	TET	AMK	GEN	TOB	FOS
14	Alcântara	14E R	SHV-12	ST3576	R	S	S	R	I	S	S	S	S	R	S	S	S	S	S	S	S
35	M. Moniz	35E R	SHV-12	ST10	R	S	S	R	I	S	S	S	S	I	S	S	S	S	S	S	S
36	M. Moniz	36E R	SHV-12	ST154	R	S	S	R	I	S	S	S	S	R	S	S	R	S	S	S	S
39	M. Moniz	39E R	CTX-M-15	ST206	R	S	I	I	I	S	S	S	S	S	S	S	S	S	S	S	S
44	Caxias	44E R	CTX-M-27	ST131	R	S	R	I	I	S	S	S	S	I	I	R	R	S	R	R	S
47	Caxias	47E R	CTX-M-15	ST2858	R	S	I	I	I	S	S	S	S	I	S	S	S	S	S	S	S
50	Caxias	50E R	CTX-M-27	ST131	R	S	I	I	I	S	S	S	S	I	S	R	R	S	R	R	S
56	Caxias	56E R	CTX-M-15	ST2858	R	S	R	I	I	S	S	S	S	I	S	S	S	S	S	S	S
60	Caxias	60E R	CTX-M-27	ST1488	R	S	R	R	I	S	S	S	S	I	I	R	R	S	S	S	S

TIC—Ticarcillin; AMC—Amoxicillin/clavulanic acid; CTX—Cefotaxime; CZD—Ceftazidime; TEM—Temocillin; FOX—Cefoxitin; ETP—Ertapenem; IMP—Imipenem; CZA—Ceftazidime/avibactam; ATM—Aztreonam; CIP—Ciprofloxacin; SXT—Trimethoprim-sulfamethoxazole; TET—Tetracycline; AMK—Amikacin; GEN—Gentamicin; TOB—Tobramycin; FOS—Fosfomycin.

## Data Availability

The data presented in this study are all available in the main text.
